# Cysteine Proteome Reveals Response to Endogenous Oxidative Stress in *Bacillus cereus*

**DOI:** 10.3390/ijms22147550

**Published:** 2021-07-14

**Authors:** Fella Hamitouche, Jean Armengaud, Luc Dedieu, Catherine Duport

**Affiliations:** 1 Biology Department, Campus Jean-Henri Fabre, Avignon University, INRAE, UMR SQPOV, CEDEX 09, 84911 Avignon, France; fella.hamitouche@univ-avignon.fr (F.H.); luc.dedieu@univ-avignon.fr (L.D.); 2 Département Médicaments et Technologies pour la Santé (DMTS), Université Paris-Saclay, CEA, INRAE, SPI, 30200 Bagnols-sur-Cèze, France; jean.armengaud@cea.fr

**Keywords:** aerobic growth, *Bacillus cereus*, proteome, cysteine, ROS

## Abstract

At the end of exponential growth, aerobic bacteria have to cope with the accumulation of endogenous reactive oxygen species (ROS). One of the main targets of these ROS is cysteine residues in proteins. This study uses liquid chromatography coupled to high-resolution tandem mass spectrometry to detect significant changes in protein abundance and thiol status for cysteine-containing proteins from *Bacillus cereus* during aerobic exponential growth. The proteomic profiles of cultures at early-, middle-, and late-exponential growth phases reveals that (i) enrichment in proteins dedicated to fighting ROS as growth progressed, (ii) a decrease in both overall proteome cysteine content and thiol proteome redox status, and (iii) changes to the reduced thiol status of some key proteins, such as the transition state transcriptional regulator AbrB. Taken together, our data indicate that growth under oxic conditions requires increased allocation of protein resources to attenuate the negative effects of ROS. Our data also provide a strong basis to understand the response mechanisms used by *B. cereus* to deal with endogenous oxidative stress.

## 1. Introduction

For aerobic bacteria, oxygen (O_2_) is an essential element that acts as the final acceptor in respiratory chain electron transport to efficiently produce energy [1]. During normal electron transport, oxygen is partially reduced to H_2_O [2], and small quantities of reactive oxygen species (ROS) are produced [3]. The superoxide anion (O_2_^•−^)—the product of one-electron reduction of oxygen—is the precursor to all the other ROS [3]. Thus, O_2_^•−^generates hydrogen peroxide (H_2_O_2_) by dismutation—either spontaneous or catalyzed by superoxide dismutase (SOD). H_2_O_2_ can subsequently be fully reduced to H_2_O, or it can react with ferrous ion (through the Fenton reaction) to generate the highly reactive hydroxyl radical (OH^•^) [3,4]. H_2_O_2_, O_2_^•^, and OH^•^ are all highly reactive molecules that can oxidize any macromolecule, including DNA, RNA, lipids, and proteins. Oxidation of any of these macromolecules can have a deleterious effect on multiple cellular processes [4,5]. To avoid these effects, bacteria have evolved mechanisms to counteract ROS production and minimize their harmful consequences. These mechanisms constitute a complex network of enzymatic and non-enzymatic detoxification systems. Among the enzymatic systems identified, many are widely distributed across aerobic bacteria (e.g., SOD, catalase, and alkyl hydroperoxide reductase). SOD is considered the first line of defense in the antioxidant system because it detoxifies O_2_^•−^ by producing H_2_O_2_. This molecule can then be further detoxified through the action of both catalase and alkyl hydroperoxide [6,7]. In parallel, the non-enzymatic antioxidant systems mainly rely on low molecular weight (LMW) thiols. These molecules function as cellular redox buffers to maintain the reduced status of the cytoplasm [8]. The most extensively characterized LMW thiol is glutathione (GSH), which is found in eukaryotes and gram negative bacteria [9]; gram positive bacteria use alternative LMW thiols, such as bacillithiol (BSH) and mycothiol (MSH) [9].

Although ROS are mainly associated with oxidative stress, they also serve as signaling molecules to regulate cellular processes [10,11]. Thus, among ROS, H_2_O_2_ has emerged as the major redox signaling metabolite, thanks to its ability to rapidly and specifically mediate the reversible oxidation of cysteine thiols in proteins [12]. Cysteine is an ideal target for modification as it can exist in several oxidation states following H_2_O_2_ oxidation: Sulfenic acid (–SOH), disulfide bond (–S–S–), and mixed disulfide bond with an LMW thiol [13]. These oxidative cysteine modifications serve as rapid switches to modulate protein function and induce signal transduction cascades [14,15]. Numerous recent reports illustrate how cysteine switches regulate intracellular processes in response to external stimuli. However, few studies have focused on cysteine-mediated redox signaling under physiological conditions, mainly because of the challenges of determining the cysteine redox status [16].

*Bacillus cereus* is a facultative aerobic foodborne pathogen; its metabolism has been extensively characterized [17]. In O_2_-regulated batch culture, *B. cereus* growth is halted once the carbon source has been exhausted and ROS accumulate [18,19]. Despite decades of intensive investigations, which unraveled several regulatory processes, environmental sensors, and repair mechanisms [17,20,21,22], we know little about the link between cellular physiology and the number and redox status of cysteine residues present in proteins.

In this study, we used a comparative, quantitative total proteomics strategy to investigate the temporal dynamics of the whole *B. cereus* proteome and its thiol proteome in aerobic batch cultures, where glucose was provided as limited carbon and energy source. We explored the thiol proteome using a differential labeling strategy, and analyzed samples by liquid chromatography-tandem mass spectrometry (LC-MS/MS), which is a powerful means to detect redox modifications on cysteine residues [23,24]. Our results revealed that *B. cereus* adjusts its proteome both at the protein and cysteine residue levels to deal with ROS, which accumulates over time during aerobic growth.

## 2. Results and Discussion

### 2.1. B. cereus Proteome Is Enriched in Antioxidant Proteins as Aerobic Growth Progresses

**Figure 1:**
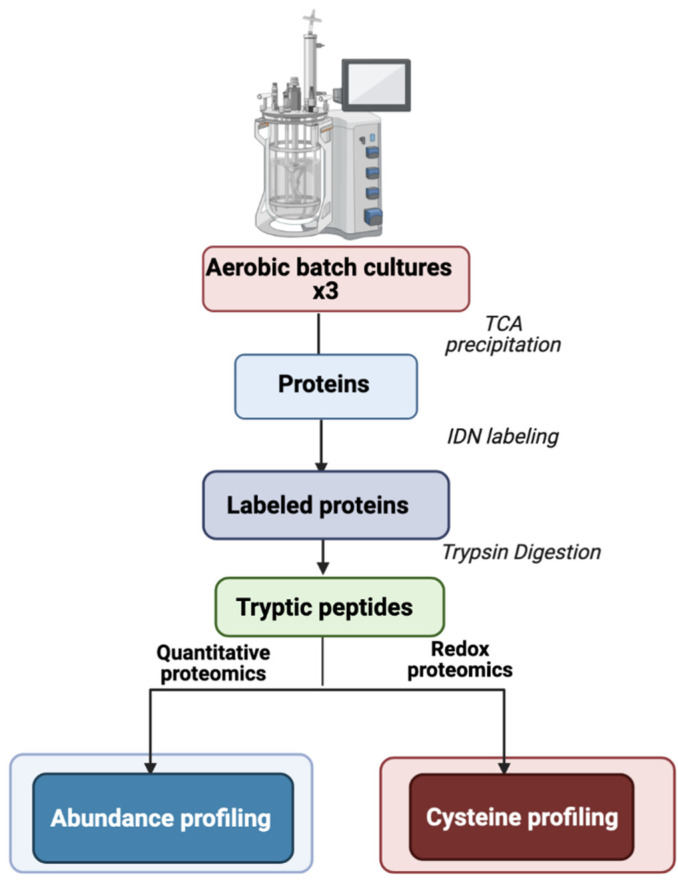
Experimental design. Protein extracts were harvested at early- (EEP), mid- (MEP), and late- (LEP) exponential growth phases. The IDN differential cysteine trapping method consisted of labeling reduced cysteines with iodoacetamide, reducing oxidized cysteines with DTT, and then labeling them with *N*-ethylmaleimide.

*B. cereus* ATCC 14579 was grown in pO_2_-regulated conditions as described previously [19]. Three independent biological replicates were collected at early (EEP), middle (MEP), and late (LEP) exponential growth phases (Figure S1). Protein extracts were prepared without separating cells from culture supernatants, in contrast to previous studies [19,21], to provide a comprehensive view of changes to the thiol proteome. Cell lysis and protein extraction were performed in TCA directly in sampling vials to acquire a snapshot of the in vivo redox status of cysteine residues [16,23]. Cysteine thiols were then labeled sequentially using a differential alkylation technique [25]. Proteins and cysteine thiol modifications were identified and quantified from tryptic peptides following LC-MS/MS analysis (Figure 1).

**Figure 2:**
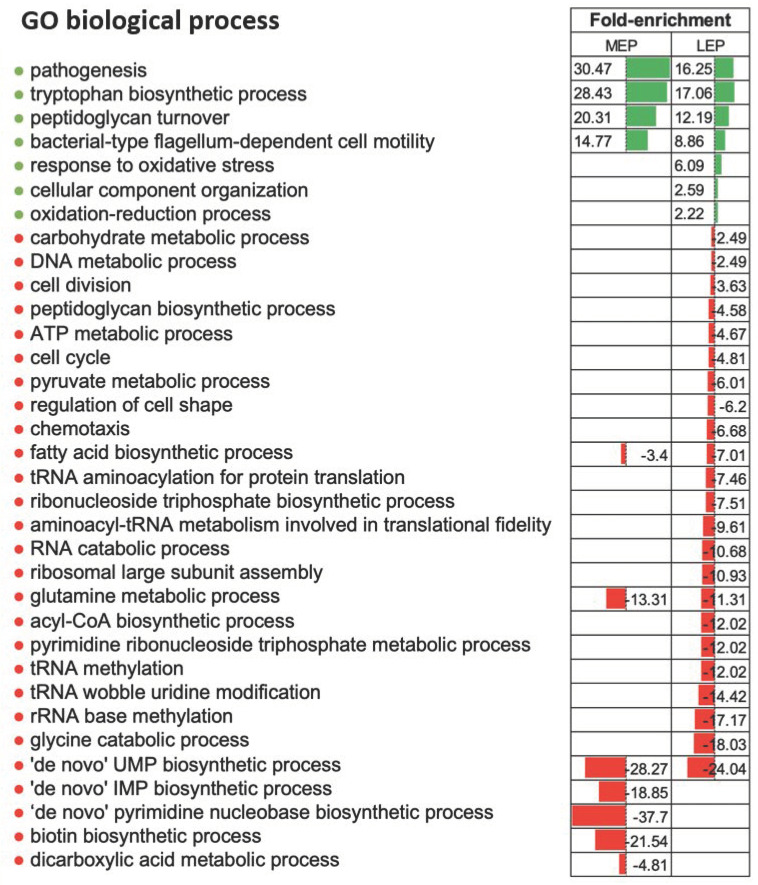
Enriched biological processes associated with differentially accumulated proteins (DAPs). Bars represent the biological processes (gene ontology) that were significantly enriched (green) or non-enriched (red) (adjusted *p*-value ≤ 0.05, Fisher’s test) at the mid-exponential growth phase (MEP) or late-exponential growth phase (LEP) compared to the early exponential growth phase.

In total, 1648 proteins were confidently identified by at least two peptides (Table S1). We compared the abundance of these proteins between the three growth stages. A protein was considered as a differentially accumulated protein (DAP) when its fold-change ratio exceeded 1.5 (|FC| ≥ 1.5) with a *p*-value ≤ 0.05. Based on these criteria, we identified 134 down- and 145 upregulated DAPs in MEP compared to EEP, and 226 down- and 224 upregulated DAPs in LEP compared to EEP. Among the DAPs identified in LEP samples, levels of 40 decreased during the transition from MEP to LEP, levels of 57 increased, and levels of 1203 were not significantly altered (Table S2). Taken together, the data obtained indicate that the *B. cereus* proteome is highly dynamic over the course of exponential growth, and that changes in protein abundances become more pronounced the longer cells are maintained in culture. Such changes during growth generally reflect sequential activation and repression of specific cellular processes. To identify which cellular processes were implicated, we performed a statistical enrichment of DAPs based on gene ontology (GO) categories corresponding to biological processes and cellular components. The number of distinct biological processes associated with DAPs between LEP and EEP was higher than the number between MEP and EEP, which is in line with the overall numbers of DAPs identified from the differential analyses (Figure 2). Several biological processes associated with primary metabolism were significantly depleted in terms of proteins when cells entered MEP (nucleotide metabolism), or LEP (translation and fatty acid metabolism). These alterations reflect reduced demand for macromolecule synthesis at later stages of growth, particularly when cultures enter the stationary phase [26,27]. Several processes associated with virulence or pathogenesis—particularly enterotoxin biosynthesis and proteolysis—and motility were significantly enriched among the proteins identified as upregulated when cells entered the MEP (Figure 2). These results are in line with previous reports [19,21]. Indeed, the corresponding biological processes have been shown to be growth phase-dependent and controlled by a variety of pleiotropic regulators [17,28].

Interestingly, we noted that proteins involved in the tryptophan biosynthesis pathway and the pathway controlling the response to oxidative stress were enriched when cells entered MEP and LEP, respectively. This enrichment most likely reflects several factors: An accumulation of ROS in the final stages of growth, the possibility that tryptophan metabolic enzymes are attractive targets for ROS, and attempts by bacteria to counteract oxidative stress to limit cellular damage [29,30]. For the latter, cells have evolved mechanisms, including repair systems, inducing cross-protection between starvation and oxidative stress, and strengthening the cell wall to maintain bacterial morphology [31,32]. Finally, *B. cereus* could adjust its proteome over the course of growth to deal with, and respond to, the gradually deteriorating conditions, which include carbon starvation and ROS accumulation [17,18,33].

### 2.2. The Cysteine Content of the B. cereus Proteome Decreases as Growth Progresses

**Figure 3:**
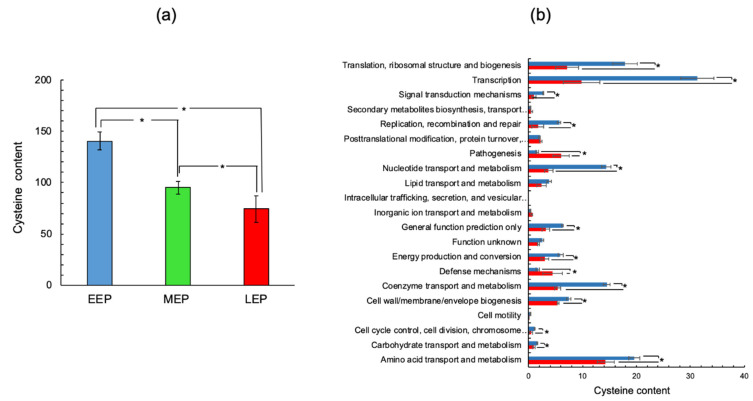
Cysteine content of *B. cereus* ATCC14579 proteome. (**a**) Overall pathway-associated cysteine abundance was determined for the early- (EEP, blue), mid- (MEP, green), and late- (LEP, red) exponential growth phases. (**b**) COG pathway-associated cysteine abundance was determined for the early- (EEP, blue), and late- (LEP, red) exponential growth phases. The cysteine abundance associated with each protein was calculated by multiplying the normalized spectral abundance factor (NSAF) value by the number of cysteine residues. Units are arbitrary units. * indicates a significant difference (Student *t*-test, *p*-value ≤ 0.05).

Although antioxidant systems are induced at the end of growth, a cysteine-rich proteome remains a prime target for ROS. To assess the cysteine content of the *B. cereus* proteome and how it changes as growth progresses, we focused on DAPs. The contribution of each DAP to the proteome’s cysteine content was calculated by multiplying the number of cysteine residues by the normalized spectral abundance factor (NSAF) value for the DAP (Table S3). Figure 3a shows that the cysteine content of DAPs tended to decrease as growth progressed; as a result, the proportion of cysteines contained in the proteome was 47 ± 11% lower at LEP than EEP. Classification of proteins into COG functional groups revealed that reduced accumulation of proteins associated with transcription, and to a lesser extent with biosynthetic pathways (nucleotide, lipid, and coenzyme metabolism) was the main reason for the decrease in cysteine content observed in the *B. cereus* proteome (Figure 3b). Indeed, proteins classified in these categories are generally cysteine-rich proteins (more than two cysteine residues per protein, Table S3). Interestingly, functional classes corresponding to pathogenesis and defense mechanisms had an increased cysteine content at LEP (Figure 3b). This increase in cysteine levels linked to pathogenesis was mainly due to the increased abundance of the associated proteins (Figure 2). Indeed, most of the proteins identified in this functional class were cysteine-poor proteins, especially the L2, B, and B’ components of Hemolysin BL (BC3101, BC3102, and BC3104: no cysteine residues), and the NheA (BC1809: one cysteine residue) and NheB (BC1810: no cysteine residues) components of non-hemolytic enterotoxin Nhe (Table S3). Enterotoxins are typically extracellular proteins, exported from the cytoplasm to the extracellular medium [34,35]. *B. cereus* could protect these proteins from both cytoplasmic [19] and extracellular oxidation [36] by excluding cysteine residues from their primary sequences. In contrast, a cysteine-exclusion approach was not applied to cholesterol-dependent hemolysin I (CLO, HlyI, BC5101), which contains three cysteine residues and is thiol-activated [37].

Unlike pathogenesis-associated proteins, most of the enzymes linked to defense mechanisms are cysteine-rich proteins—including alkyl hydroperoxide reductase subunit F (BC0376, five cysteine residues), and glutathione peroxidase (BC2114, three cysteine residues). The cysteine residues contained in these proteins are essential to their antioxidant activity [38]. In summary, our data indicated that *B. cereus* decreases the cysteine content of its proteome by decreasing the abundance of cysteine-rich proteins as growth progresses, except for antioxidant proteins, which play crucial roles in the later stages of growth. By decreasing the cysteine content of its proteome, *B. cereus* may be attempting to avoid oxidative damage to cysteine residues through their thiol functional groups.

### 2.3. The Thiol Reduction Status of the B. cereus Proteome Decreases as Aerobic Growth Progresses

**Figure 4:**
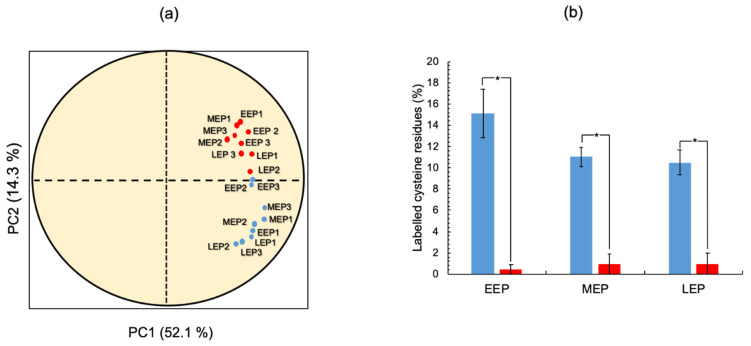
Temporal dynamics of *B. cereus* ATCC14579 thiol proteome. (**a**) Individuals factor map (PCA). PCA was carried out from a dataset, including 18 individuals (3 replicates × 3 protein samples harvested at early- (EEP), mid- (MEP), and late- (LEP) exponential growth phases x 2 cysteine residue labeling) and 286 variables (proteins). The plot shows the projection of iodoacetamide (IAM, blue) and *N*-ethylmaleimide (NEM, red)-labeled individuals in the PC1 and PC2 plane. PC2 opposes IAM and NEM-labeled individuals. (**b**) Temporal changes to non-redundant IAMCys (blue) and NEMCys (red) proteins. * indicates a significant difference (Student *t*-test, *p*-value ≤ 0.05).

To understand the full extent of oxidative damage incurred during aerobic growth, it is essential to comprehensively identify protein thiols. To do so, we applied the approach we developed previously [25]. Briefly, we tagged the thiol groups of cysteine residues by differential alkylation: Iodoacetamide (IAM) was used to label endogenously reduced cysteine thiols (IAMCys), whereas *N*-ethylmaleimide (NEM) was used to label endogenously oxidized cysteine thiols (NEMCys) following their reduction with DTT [39]. In total, we detected 1886 ± 278, 1339 ± 28, and 1190 ± 286 labeled cysteine residues at EEP, MEP, and LEP, respectively. These residues were included in a total of 746 ± 94, 559 ± 2, and 479 ± 95 non-redundant (unique) cysteine-containing peptides at EEP, MEP, and LEP, respectively (Table S4). To ensure robust analysis, we focused our analysis on the labeled cysteine residues detected in all three replicates for each growth stage. Applying these criteria, we obtained a dataset comprising 462 labeled cysteine residues derived from 286 proteins (Table S5). Principal component analysis (PCA) revealed that these 286 proteins have distinguishable IAMCys and NEMCys profiles (Figure 4a). We, therefore, analyzed the growth phase-dependent changes to IAMCys and NEMCys levels separately across the 286 proteins. Figure 4b shows that the total number of NEMCys remained constant throughout growth, whereas the number of total IAMCys decreased as growth progressed. In addition, LEP was found to be associated with a lower IAMCys/NEMCys ratio than MEP and EEP, indicating that the oxidation status of the *B. cereus* thiol proteome had increased at the end of exponential growth.

#### 2.3.1. Temporal Dynamics of NEMCys-Labeled Proteins (NEMCys Proteins)

Filtering for NEMCys proteins detected in all replicates for at least one growth phase identified 87 distinct proteins, of which 15 showed significant changes in NEMCys levels at MEP and LEP compared to EEP (*p* ≤ 0.05, |FC| ≥ 1.5) (Table S6). LEP extracts contained a higher number of proteins with increased NEMCys levels (5 proteins) than MEP (2 proteins). However, among these proteins, only inosine -5′-monophosphate dehydrogenase (GuaB) had an increased NEMCys level not associated with an increase in protein abundance, indicating a higher oxidation status at LEP than EEP (Table S6). GuaB is a key component in the purine biosynthesis pathway, which begins with the conversion of the intermediate of the phosphate pentose pathway (PPP), ribose-5 phosphate, to inosine monophosphate (IMP), and its subsequent metabolism to produce guanosine and adenine monophosphate (GMP and AMP) [40]. GuaB has a redox-sensitive thiol in its active site (Cys^308^) in *B. subtilis* [41], and has been reported to be S-thiolated in an oxidative environment [36].

Considering the NEMCys proteins as sole variables reflect neither the thiol proteome’s redox status nor its temporal dynamics. Nevertheless, the low level of NEMCys proteins results from the low level of proteins containing oxidized thiols present in reduced cytoplasm, thanks to the efficiency of the cell’s antioxidant systems [42,43].

#### 2.3.2. Temporal Dynamics of IAMCys-Labeled Proteins (IAMCys Proteins)

**Figure 5:**
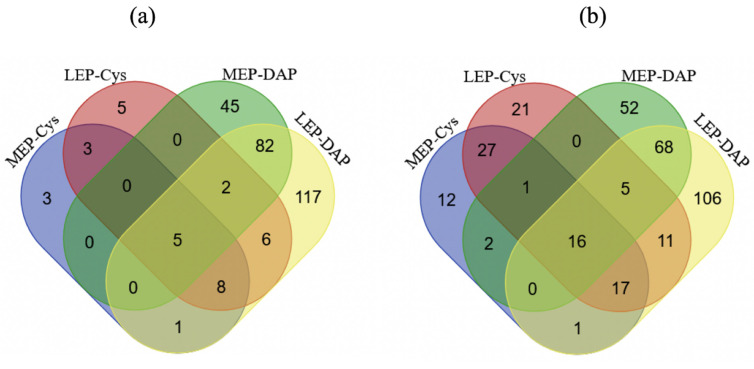
Venn diagrams showing proteins found to be upregulated (**a**) and downregulated (**b**) in terms of abundance (DAP) and iodoacetamide labeled cysteine residue (Cys) levels at mid- (MEP) and late- (LEP) exponential growth phases.

We identified 278 IAMCys proteins in all three replicates for at least one growth phase. The striking difference between the number of IAMCys proteins (278) and NEMCys proteins (87) indicates that the most abundant proteins in cells tend to have reduced thiols, whatever the growth phase. Of the IAMCys proteins, 146 were found to differentially accumulate IAMCys residues as growth progressed: IAMCys levels were decreased for 113 proteins in LEP compared to MEP, whereas IAMCys levels were increased for 33 proteins (Table S7). From among these proteins, we identified redox-sensitive proteins, i.e., proteins for which the observed differences in the level of IAMCys were not linked to any difference in protein abundance (Figure 5).

Of the 71 proteins identified, 11 had significantly increased IAMCys levels (Figure 5a), and 60 had significantly decreased IAMCys levels (Figure 5b). We noted that, in comparison to EEP, LEP was associated with a higher number of proteins with decreased IAMCys proteins (48 proteins) than MEP (39 proteins). If we consider the relative change in IAM modification within a protein to be a readout for cysteine thiol oxidation [39], our results indicate that increased numbers of proteins are subjected to thiol oxidation than thiol reduction as growth progresses. Proteins sensitive to growth phase-dependent thiol oxidation were mainly associated with primary metabolic processes, such as translation or amino acid, carbohydrate, nucleotide, lipid, and energy metabolism processes (Table 1). The translation machinery includes ribosomal proteins and elongation factors (EF). The ability of bacterial EF-Ts to bind EF-Tu and catalyze GDP-GTP exchange depends on the thiol group for Cys^22^ [44]. We found Cys^22^ to be significantly less modified by IAM in LEP cultures compared to in EEP cultures (Table 1), indicating a thiol defect at the end of exponential growth. This defect could mediate a direct, and thus, rapid decrease in translation [45].

Among the proteins classified as related to nucleotide metabolism, we identified GMP synthase GuaA (BC0296) as a redox-regulated protein. Like GuaB, the enzyme GuaA is involved in purine metabolism, but also in early morphogenic steps in cytokinesis, and it can be inactivated by oxidants [46].

Energy metabolism refers to metabolic pathways involved in ATP synthesis linked to NADH turnover [47]. It comprises metabolic pathways producing ROS, as well as metabolic pathways targeted by oxidant species. Among the key oxidant targets, we identified glyceraldehyde-3-phosphate dehydrogenase, tricarboxylic acid enzymes, and succinate dehydrogenase [48] as redox-sensitive proteins in our study (Table 1) alongside dihydrolipoamide dehydrogenase (DLDH, BC3970). DLDH is a flavoprotein that catalyzes the NAD^+^-dependent oxidation of dihydrolipoamide in a number of protein complexes. It can serve as the E2 subunit in the pyruvate dehydrogenase complex, converting pyruvate into acetyl-CoA to link glycolysis to the TCA cycle [49]. DLDH has two redox-sensitive cysteine residues in its active center (Cys^47^ and Cys^52^) that are indispensable for its catalytic function [50]. These two Cys residues are separated by four amino acid residues (CLNVGC in *B. cereus*) and were significantly more labeled with IAM at EEP than at later growth stages (Table 1), suggesting that they are vulnerable to oxidative inactivation [51,52]. Redox-dependent inactivation of DLDH could help cells to reroute metabolism at the end of growth to deal with starvation, and to limit ROS production, since DLDH can be a source of ROS [53]. Interestingly, in human mitochondria, inhibition of DLDH is reported to induce antioxidant responses [54]. Proliferating bacterial cells must coordinate energy metabolism with the cell cycle and cell shape [55], possibly through redox regulation, and we effectively-identified redox-sensitive proteins in all three functional classes.

Post-translational modification and activation of chaperones are part of strategies developed by bacteria to protect proteins against oxidative damages [56]. DnaK and DnaJ are components of the DnaK/DnaJ/GrpE chaperone system involved in refolding proteins to their native conformation [57]. Unlike Hsp33, which transfers client proteins to DnaK/DnaJ/GrpE, the chaperone activity of DnaK and DnaJ is not reported to be oxidation-dependent [58], even though these proteins contain redox-sensitive thiols (Table 1).

In contrast, several transcriptional factors are known to be redox-sensitive, and required to counteract oxidative stress [59]. Here, we identified AbrB, with its single Cys^54^ residue, as a redox-sensitive transcriptional regulator. AbrB is a crucial transcription state regulatory protein that represses the expression of stationary phase-specific genes during the exponential growth of *B. subtilis* cells [60,61], as well as repressing the synthesis of virulence factors by *B. cereus* [62,63]. AbrB can only bind DNA to fulfill its regulatory activity if its Cys^54^ residue is in a non-oxidized state. It has been suggested that the free sulfhydryl group on Cys^54^ is important for the structure and stability of the active form of the native protein [64]. Therefore, the repressive effect of the free thiol group could decrease as growth progresses.

## 3. Materials and Methods

### 3.1. B. cereus Growth Conditions

*B. cereus* ATCC14579 batch culture was carried out in a Bioreactor (My-control, Applikon technology, equipped with Mettler Toledo Dissolved oxygen and pH probes) containing 1.5 L of MOD medium supplemented with 30 mM glucose [19,65]. Culture medium was inoculated at an OD_600_ of 0.03 from overnight preculture. The medium was adjusted to pH 7 with KOH and HCl; temperature was maintained at 37 °C. Oxygen was maintained at pO_2_ close to 20% via a sequential cascade control of airflow rate and agitation speed. Cultures were performed in triplicates.

### 3.2. Protein Extraction for Proteome Analysis

Samples were harvested at three time-points: Early exponential growth phase (EEP, t = 1.5 h, OD_600nm_ = 0.2), mid-exponential growth phase (MEP, t = 3.5 h, OD_600nm_ = 1.5), and late-exponential growth phase (LEP, t = 4.5 h, OD _600nm_ = 3). Cell lysis, and protein extraction were performed in TCA vials containing 180, 45, and 45 mL EEP-, MEP- and LEP- samples, respectively, as previously described [25]. Extracted proteins were denatured with DAB buffer (6 M urea, 200 mM Tris-HCl pH 8.5, 10 mM EDTA, and 2% *w*/*w* SDS) and adjusted to pH 7. Subsequently, samples were labeled by a differential thiol trapping method. The thiol labeling strategy, named IDN, is described in detail elsewhere [25]. Briefly, samples were first labeled with 50 mM iodoacetamide (IAM) to alkylate reduced cysteine residues, then treated with 25 mM DTT to reduce oxidized cysteine residues before labeling DTT-reduced cysteine residues with 150 mM *N*-ethylmaleimide (NEM). The efficiency of the IAM alkylation step was assessed for each sample by IN labeling, i.e., without application of DTT between IAM and NEM labeling. This control generated a dataset that was used to subtract cysteine residues that had escaped IAM labeling from the NEM dataset. Protein concentration was determined using a BCA protein assay kit (Pierce) as recommended by the supplier, and a total of 100 µg of proteins was used for the subsequent protein digestion.

### 3.3. Protein Identification and Quantification

Proteins were digested in-gel with sequencing grade, modified trypsin, as previously described [66]. The digested peptides were separated on an ultimate 3000 nano LC system coupled to a Q-Exactive HF mass spectrometer (ThermoFisher Scientific, llkirch-Graffenstaden, France) for analysis. Peptide mixtures (10 µL) were loaded, desalted online, and then resolved according to their hydrophobicity on a nanoscale Acclaim Pepmap 100 C18 column (3-μm bead size, 100-Å pore size, 15 cm × 75 μm) at a flow rate of 200 nL/min using a bi-modal 120-min gradient combining buffer B (0.1% HCOOH, 80% CH_3_CN, 20% H_2_O) and buffer A (0.1% HCOOH, 100% H_2_O): 4–25% B in 100 min, followed by 25–40% B in 20 min. Mass spectrometry was performed in data-dependent acquisition mode following a Top20 strategy with full MS scans acquired from 350 to 1800 *m/z* at 60,000 resolution. After each scan, the 20 most abundant precursor ions were sequentially selected for fragmentation and MS/MS acquisition at 15,000 resolution. An intensity threshold of 9 × 10^4^ was applied. A 10-s dynamic exclusion was used to increase the detection of low-abundance peptides. Only double- and triple-charged ions were selected for MS/MS analysis.

The MS/MS data were searched against the theoretical *B. cereus* ATCC14579 annotated theoretical proteome (5216 sequences) using Mascot software, version 2.5.1 (Matrix Science). The search parameters included adherence to the enzymatic cleavage rule for trypsin, only 2^+^ and 3^+^ peptide charges, 5 ppm mass tolerance for the parent ion, 0.02 Da mass tolerance for MS/MS ions, and a maximum of two missed cleavage sites. For the database search, only the following variable modifications were considered: *N*-ethylmaleimide (C), carbamidomethyl (C), carbamyl (K), oxidation (M), deamidation (NQ). All peptide matches with a peptide score associated with a Mascot *p*-value of less than 0.05 were retained. Proteins were considered valid when at least two distinct peptides were detected in the same sample. The mass spectrometry proteomics data have been deposited to the ProteomeXchange Consortium via the PRIDE partner repository under dataset identifiers PXD025504 and 10.6019/PXD025504.

### 3.4. Bioinformatics Analysis

Only proteins identified by at least two distinct peptides were regarded as present and reliably identified in samples. The Limma package for R was used to quantify proteins and analyze statistical significance. Proteins for which a fold-change (FC) ≥ |1.5| and *p*-value < 0.05 was detected at one growth stage compared to the other growth stages were designated as differentially abundant proteins (DAPs). FC values were Log_2_-transformed to facilitate further analysis. Quantification of labeled cysteine-containing peptides was restricted to peptides identified in all three replicates for any individual sample. Gene Ontology enrichment analysis was performed using the Panther Gene Ontology tool (http://geneontology.org/, accessed on 2 July 2021). The FactomineR package for R was used for principal component analysis (PCA). PCA was carried out with biological replicates of each growth phase as individuals and the IAMCys and NEMCys values assigned to proteins as quantitative variables. Principal components 1 (PC1) and 2 (PC2) define the plane that maximizes the variance of the individuals when projected into a two-dimensional space. Each PC defines a proportion of the total variance, with PC1 explaining the greatest amount of variation. Applied to our data of 18 individuals, PC1 and PC2 explained 52.1% and 14.3% of the total variation, respectively, and separate NEM-individuals from IAM individuals. In silico analysis for cysteine composition in the identified proteome was performed by calculating the cysteine occurrence on sequences from data files created by the Uniprot database. The Venn diagram was drawn with Draw Venn Diagram (http://bioinformatics.psb.ugent.be/webtools/Venn/, accessed on 2 July 2021).

## 4. Conclusions

**Figure 6:**
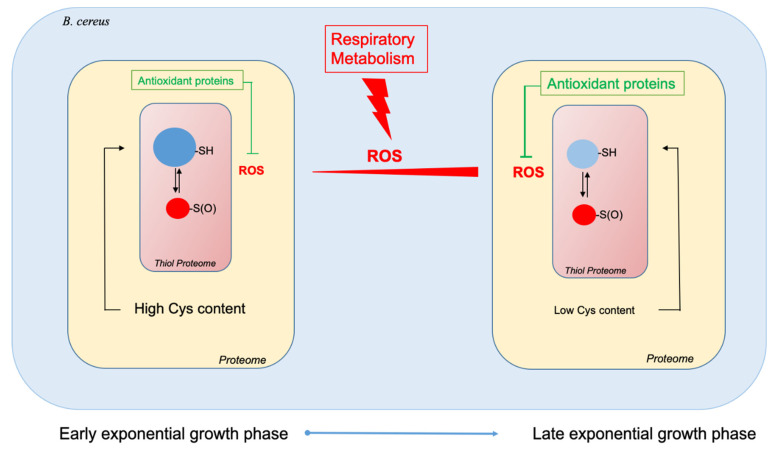
Schematic representation of the *B. cereus* ATCC14579 proteome’s remodeling between the beginning of the exponential growth phase and the end of the exponential growth phase to cope with endogenous ROS production.

Aerobic bacteria must deal with endogenous ROS, which are mainly generated as by-products of respiratory metabolism. To minimize the damage caused by these molecules, bacteria can implement various strategies [4,67]. Our proteomics analysis showed that *B. cereus* increases the production of proteins with functions related to oxidative stress and detoxification of ROS as aerobic growth progresses. Among these antioxidant proteins, tryptophan metabolic enzymes could be key elements [68,69,70]. Enrichment of the proteome in antioxidant proteins at the end of exponential growth is accompanied by a depletion of cysteine-rich proteins, in particular enzymes required for primary metabolism. This overall change results in a decrease in the proportion of cysteine residues in the proteome by approximately 47%, and could contribute to maintaining a healthy proteome, since cysteine residues are highly sensitive to ROS [38]. However, cysteine proteome remodeling does not allow the thiol proteome to entirely maintain its reduced status during exponential growth (Figure 6). Consequently, the strategy implemented by *B. cereus* at the proteome level to cope with ROS may not be sufficient to maintain the redox status of the thiol proteome.

Proteome remodeling is orchestrated by several pleiotropic regulators, including the transition state regulator AbrB [71]. Our results indicate that the thiol reduction status of AbrB changes as growth progresses. Although previous reports suggested a possible redox regulation of AbrB’s DNA binding activity, further studies will be needed to confirm that the thiol reduction status observed here effectively plays a regulatory role.

Finally, the ability of aerobic bacteria to cope with endogenous ROS may originate from their capacity to modulate their cysteine proteome, as observed in *B. cereus*.

## Figures and Tables

**Table 1:** Proteins with differentially iodoacetamide (IAM) labeled cysteine residues at mid-exponential growth phase (MEP), and late-exponential growth phase (LEP) compared to early exponential growth phase (EEP).

Gene ID	Protein	IAM Labeled Cys Peptide	Log_2_(FC) *	
			MEP	LEP
Proteins with increased levels of IAMCys residues				
Transcription				
BC4603	Transcriptional regulator, GntR family	IVC^113^NLPK	1.0	
Translation, ribosomal structure, and biogenesis				
BC0155	50S ribosomal protein L36 (RpmJ)	VRPSVKPIC^11^EK		2.2
		GKVMVIC^27^ENPK		
Signal transduction mechanisms				
BC0442	Tellurium resistance protein (TerD)	LSTC^79^GSIIHSGDNLTGEGAGDDETIFVELHK		1.6
		LVFVVNIYDC^123^VNR		
Secondary metabolite biosynthesis, transport, and catabolism				
BC2305	Isochorismatase (DhbB)	C^197^AVTTSTNLLLK		1.7
		C^67^KELGIPVVYTAQPGGQTLEQR		
Coenzyme transport and metabolism				
BC4853	1,4-dihydroxy-2-naphthoyl-CoA synthase (MenB)	EIWYLC^174^R	1.0	1.6
BC1086	Lipoate--protein ligase	AFC^71^SGGDQKVR	1.6	
		INLAIEEYC^274^VK		
Post-translational modification, protein turnover, chaperones				
BC0517	Thioredoxin-dependent thiol peroxidase	DMTPGC^47^TTEAC^52^DFR		2.0
Amino acid transport and metabolism				
BC1182	Oligopeptide transport ATP-binding protein (OppD)	VVIAMALAC^172^NPK	1.7	1.2
Function unknown				
BC5199	Xaa-Pro dipeptidase	FIC^25^YISR	1.6	1.1
BC4341	GTP pyrophosphokinase	ITC^88^C^89^FVEDIYHLK		
BC3302	Pentapeptide repeat containing protein	SC^78^NLEEIHIADC^88^R	1.8	2.1
		ASFFDC^68^DFEFADFR		
Proteins with decreased levels of IAMCys residues				
Amino acid transport and metabolism				
BC1740	Aspartate ammonia-lyase	AFTDNC^393^LK		−0.8
BC1546	Aminotransferase	DQGIAYDPSEIIVC^96^NGAK		−1.3
BC0055	D-alanyl-D-alanine carboxypeptidase (DacA)	TGSTPEAGDC^169^FTGTVER		−1.0
BC4981	Cysteine desulfurase (SufS)	VDVQDLNC^215^DFYALSAHK		−1.3
		MC^226^GPTGIGVLYGK		
		AGHHC^361^AQPLMK		
BC3798	Aspartokinase (DapG)	HLQTVTYNEIC^206^NMAYQGAK	−2.6	−2.1
		LLQNLGYEPIVTEHC^339^AK		
BC4936	Diaminopimelate epimerase (DapF)	GPAEVIC^278^R	−1.0	
BC0747	Glycine oxidase (ThiO)	IENNKVTGVITSEGIVTC^201^EK	−2.1	−1.7
BC3799	Aspartate-semialdehyde dehydrogenase (AsdB)	KIMHMPELEVAATC^244^VR	−1.3	−1.8
Energy production and conversion				
BC3970	Dihydrolipoyl dehydrogenase	GIIEIDEQC^303^R		−1.7
		ANLGGVC^47^LNVGC^52^IPSK		
		VAVEAISGHASAIDYIGIPAVC^353^FTDPELASVGYTK		
BC4157	Dihydrolipoamide acetyltransferase component of pyruvate dehydrogenase complex	VLDGLIC^417^GK		−1.1
		DMVNLC^404^LSLDHR		
BC2826	Probable manganese-dependent inorganic pyrophosphatase (PpaC)	NPDTDAIC^18^SAIAYAELKK		−0.9
		SPTC^158^TEQDVAAAR		
		C^113^EPVGC^118^TATILNK		
BC4980	IscU protein	C^127^ATLAWK		−1.4
		NHGVLEDSVTVNLNNPTC^40^GDR		
BC3773	Pyruvate synthase	AAANVGLNPDELAVISGIGC^50^SGR		−2.4
		NSVKPNWCPGC^16^GDFSVQAAIQR		
BC3616	Aconitate hydratase	VVEEYC^346^K	−1.0	
BC3833	Succinate--CoA ligase subunit alpha (SucD)	LLGPNC^124^PGVITPDEC^133^K	−1.2	
		IKTMEAC^269^GIK		
		GLFETC^297^K		
BC5387	Phosphate acetyltransferase	GC^306^NEEEVYK	−1.7	−1.9
		EEEKYVFADC^167^AINIAPNSQDLAEIGIESAK		
BC4517	Succinate dehydrogenase flavoprotein subunit	EIFDVC^295^VEQKAMC^185^EAAPGIIHLMDR	−1.4	−1.8
		AVVDDEGVC^170^R		
BC4604	Malate dehydrogenase	LMEPTFGGVNLEDIAAPNC^141^FIIEER	−2.1	−2.5
		DLSLAYSPGVAEPC^45^KEIYDDKSK		
		DIIMC^215^DR		
BC4158	2-oxoisovalerate dehydrogenase beta subunit	SNNDWTC^111^PVTIR	−1.1	−1.1
Carbohydrate transport and metabolism				
BC4599	Pyruvate kinase (Pyk)	IVC^8^TIGPASESIEKLEQLIEAGMNVAR		−2.8
		C^265^NVLGKPVITATQMLDSMQR		
BC5135	Enolase (Eno B)	TGLTSAEMVDFYEELC^279^K		−2.3
BC5140	Glyceraldehyde-3-phosphate dehydrogenase (Gap1)	GILGYSEEPLVSIDYNGC^145^TASSTIDALSTMVMEGNMVK	−1.6	−1.5
BC5335	Fructose-bisphosphate aldolase (Fba)	NVSVEAELGTVGGQEDDVIAEGVIYADPAEC^161^K	−1.2	−1.3
		C^92^KEAIDAGFTSVMIDASHHPFEENVETTK		
		HLVEATGIDC^172^LAPALGSVHGPYK		
BC4571	Deblocking aminopeptidase	IGC^189^AIAIDVLK		−1.1
BC5318	Ribose 5-phosphate isomerase (RpiB)	GILVC_66_GTGIGMSIAANK	−2.0	−2.1
		C^84^ALVHDTFSAK		
BC4600	ATP-dependent 6-phosphofructokinase (PfkA)	C^283^VGIQDNK	−2.0	−1.4
Coenzyme transport and metabolism				
BC5413	Phosphomethylpyrimidine kinase (ThiD)	GADEALHPETNDC^126^LR		−1.3
BC0621	Putative pyridoxal phosphate-dependent acyltransferase	SRPFLFSTALTPADAAAC^284^MR	−1.1	
BC4468	Glutamate-1-semialdehyde 2,1-aminomutase 2 (HemL2)	VAYNC^251^GQGYYGVTPDLTC^265^LGK	−1.6	
BC4111	Riboflavin biosynthesis protein (RibBA)	GLVC^56^VPITEGYAER	−1.8	−1.6
		VPDLIEC^179^AK		
Uncategorized				
BC3977	Ribonuclease J (RnjB)	VVILC^297^TGSQGEPMAALSR		−2.5
		MAEIGKDGVLC^191^LLSDSTNSEVPNFTMSER		
BC4425	Hypothetical transcriptional regulator	VIVC^84^QHKPAEVR		−0.9
		EKLDAAC^169^EALDK		
BC3854	Predicted kinase related to hydroxyacetone kinase	DTEIDGVAIQKDDFMC^280^IADGK		−2.3
		YGYC^244^TEFMVK		
	DUF3797 domain-containing protein	TLYYVQC^20^PVC^23^K		−0.9
BC0049	Protein (SspF)	LC^133^GATPVFVDVR	−1.3	−1.0
Nucleotide transport and metabolism				
BC4402	Adenine phosphoribosyltransferase (Apt B)	GFIIGC^64^PVSYALEVGFAPVRK		−1.4
BC0296	GMP synthase (GuaA)	VLC^225^ALSGGVDSSVVAVLIHK		−1.1
		AIGDQLTC^250^IFVDHGLLR		
		GIIFSGGPNSVYGEGALHC^72^DEK		
BC5315	Uracil phosphoribosyltransferase (UppB)	LMC^159^IVAAPEGVK	−1.3	
BC0331	Phosphoribosylformylglycinamidine cyclo-ligase (PurM)	GISEGC^88^R	−1.1	−1.2
Translation, ribosomal structure, and biogenesis				
BC0153	Methionine aminopeptidase (Map)	SLVAQC^231^EHTVVVTR		−1.9
		LC^121^QAAVDAFWAAMK		
BC0144	30S ribosomal protein S14 type Z (RpsZ)	C^27^GRPHSVYR	−2.4	
BC4391	tRNA-specific 2-thiouridylase (MnmA)	KDSTGIC^200^FIGER	−1.4	
		ILC^330^DEPIR		
BC3824	Elongation factor Ts (EF-Ts)	FFEEIC^239^LLDQAFVKNPDMK	−1.7	−1.4
		EKTGAGMMDC^22^KK		
BC0153	Methionine--tRNA ligase 2 (MetG2)	VIC^506^VTNLKPVK		−1.9
		SWESLSTIGC^617^IPAGTK		
BC0352	Aspartyl/glutamyl-tRNA (GatB)	SIIQYTGVSDC^182^K	−1.2	−2.8
		C^191^DANISLRPVGQEK		
		AAMALNC^68^EIATETK		
BC0108	Glutamate-tRNA ligase (GltX2)	C^108^YMTEEELEAEREGQIAR	−1.7	−1.6
BC3923	50S ribosomal protein L32 (RpmF)	VC^42^KAC^45^GTYK	−1.5	−1.9
Cell wall/membrane/envelope biogenesis				
BC0054	Bifunctional protein (GlmU)	EINTGTYC^175^FDNK		−1.4
BC4444	Rod shape-determining protein (MreD)	TITVC^240^SEEITEALKENAAVIVQAAK	−3.2	−3.3
		ILIC^101^C^102^PTNITSVEQK		
BC0257	D-alanine-D-alanine ligase B (DdlB)	LGYPC^180^FVKPANLGSSVGINK	−1.1	−1.7
		C^239^SVVGEIVPK		
Transcription				
BC0042	Transcription state regulatory protein (AbrB)	YKPNMTC^54^QVTGEVSDGNLSLAEGK		−2.3
Lipid transport and metabolism				
BC2302	2,3-dihydro−2,3-dihydroxybenzoate dehydrogenase	C^182^NLVSPGSTETEMQR		−1.6
BC4276	4-hydroxy-3-methylbut-2-en-1-yl diphosphate synthase (IspG)	VAVLGC^303^AVNGPGEAR	−1.6	
		RLEEAGC^54^QVVR		
		SFGLASNAATLISC^268^PTCGR		
BC0353	DAGKc domain-containing protein	LEQAGYETSC^38^HATTGPGDATVAAR	1.0	
		SIEEAADIIC^116^EGK		
Cell cycle control, cell division, chromosome partitioning				
BC4442	Cell division inhibitor (MinD)	QDYDYILIDC^120^PAGIEQGFK		−1.2
		C^68^RLPQALIK		
BC4446	Cell shape-determining protein (MreB)	MPVLVAEDPLDC^319^VAIGTGK	−1.0	−1.4
		KPYVMVC^108^VPSGITAVER		
Post-translational modification, protein turnover, chaperones				
BC4312	Chaperone protein (DnaK)	SKIIGIDLGTTNSC^15^VAVMEGGEPK	−0.9	
BC4311	Chaperone protein (DnaJ)	HC^192^SGSGQVSVEQNTPFGR	−2.5	−2.2
		ELNVEIPVEDPC^146^DTC^149^K		
Intracellular trafficking, secretion, and vesicular transport				
BC3845	Signal recognition particle receptor (FtsY)	KVDVLLC^208^DTAGR	−1.1	−1.1
Secondary metabolite biosynthesis, transport, and catabolism				
BC1372	D-alanine--D-alanyl carrier protein ligase (DltA)	SLPVGYC^324^K	−2.9	
		TFLFC^269^GEVLPNEVAR		
		AC^421^SYVEGAVIVPIKK		

* Only fold-changes (FC) equal or higher than |1.5| with *p* < 0.05 are indicated.

## Data Availability

The mass spectrometry proteomics data have been deposited to the ProteomeXchange Consortium via the PRIDE partner repository under dataset identifiers PXD025504 and 10.6019/PXD025504.

## Supplementary Materials

The following are available online at https://www.mdpi.com/article/10.3390/ijms22147550/s1.
