# Supplementary figures and images for: Cysteine Proteome Reveals Response to Endogenous Oxidative Stress in Bacillus cereus
Source: Int J Mol Sci. 2021 Jul 14;22(14):7550. doi: 10.3390/ijms22147550 (PMC8305198; doi:10.3390/ijms22147550)

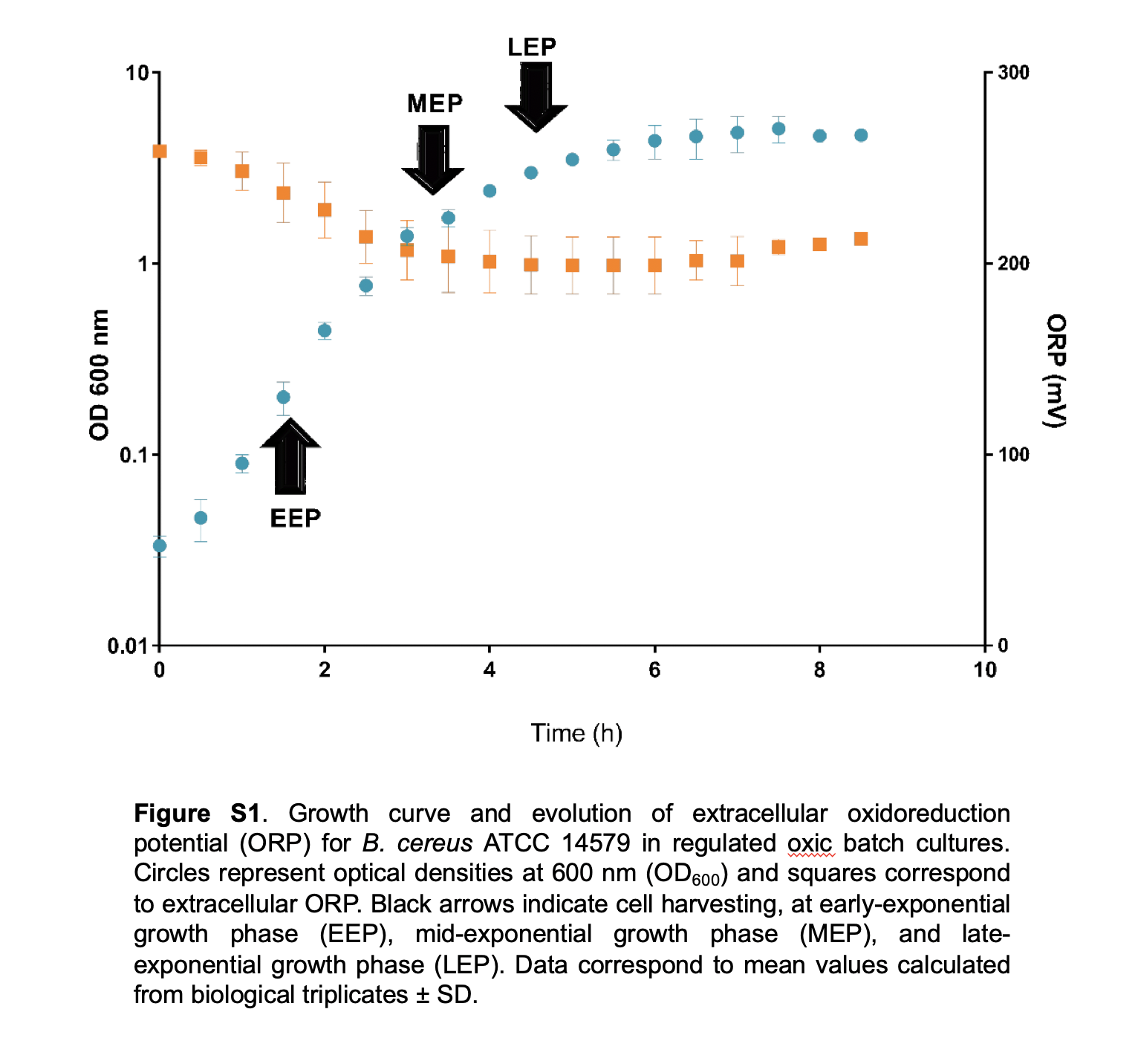

Supplement: Supplementary file 1 [file ijms-22-07550-s001.zip › Figure S1.tiff]
